# Seroprevalence of cytomegalovirus among pregnant women in Singapore

**DOI:** 10.1186/s41182-024-00634-z

**Published:** 2024-10-03

**Authors:** Pamela Partana, Wei Yee Wan, Xin Yu Venessa Chow, Jerry Kok Yen Chan, Lay Kok Tan, Wei Ching Tan, Piea Peng Lee, Gek Hsiang Lim, Liying Yang

**Affiliations:** 1 Department of Obstetrics and Gynaecology, Singapore General Hospital, Singapore, Singapore; 2 Department of Microbiology, Singapore General Hospital, Singapore, Singapore; 3 Department of Reproductive Medicine, KK Women’s and Children’s Hospital, Singapore, Singapore; 4 Division of Surgery and Surgical Oncology, National Cancer Centre Singapore, Singapore, Singapore; 5 Health Service Research Unit, Singapore General Hospital, Singapore, Singapore

**Keywords:** Cytomegalovirus, Seroprevalence, Antenatal, Singapore

## Abstract

**Background:**

Cytomegalovirus (CMV) is the most common congenital infection in pregnancy with potential long-term adverse effects on the fetus. There is limited data on CMV seroprevalence in pregnant women in Singapore, with last reported study dating back over two decades. We look at the latest CMV seroprevalence in antenatal population in Singapore.

**Methods:**

Between January 2021 and August 2021, 385 pregnant women receiving antenatal care at Singapore General Hospital were randomly selected for CMV IgG test to be performed on their blood samples collected during the first trimester of their pregnancies. Positivity for CMV IgG represents past exposure prior to pregnancy.

**Results:**

Overall CMV seroprevalence was 71.7% (276/385) (95% CI 067, 0.76, *p* value < 0.001). The trend of CMV IgG positivity increased with age, 68.3% (95% CI 0.60, 0.76, *p* value < 0.001) in those aged 20–29, 72.5% (95% CI 0.66, 0.78, *p* value < 0.001) in the 30–39 age group, and 79.0% (95% CI 0.67, 0.76, *p* value 0.012) in women over 40.

**Conclusions:**

There is a declining trend in CMV seroprevalence among pregnant women in Singapore, which indicates that a substantial portion of this population faces the risk of primary maternal CMV infection during pregnancy. Emerging research suggests that prenatal treatment with valacyclovir effectively reduces the likelihood of vertical transmission. Considering this evidence, it is imperative to reevaluate the recommendations for universal maternal CMV screening during pregnancy.

## Introduction

Cytomegalovirus (CMV; *Herpesvirus 5*) is the most common congenital infectious condition in pregnancy, with a prevalence of up to 6% of live births in low- and middle-income populations and 0.3–0.7% in high-income populations [1–7]. This ubiquitous virus typically leads to asymptomatic or mild, self-limiting infection in immunocompetent children and adults. However, when CMV in maternal circulation crosses the placenta and infects the immunologically immature fetus, it can lead to long-term permanent sequalae, including hearing loss, visual impairment, neurodevelopmental disability, cerebral palsy, and death [2, 8–10]. CMV is the leading cause of non-hereditary sensorineural hearing loss in childhood, accounting for up to 23% of all cases with profound permanent childhood hearing impairment [9].

Just like other members of the Herpesviridae family, CMV remains latent in human cells following primary infection, enabling episodes of reactivation with viral replication in seropositive individual. Both primary and non-primary CMV infections during pregnancy can lead to fetal infection. However, the greatest risk to the fetus mainly occurs in primary acquisition of the virus during pregnancy (maternal primary infection), where there is a fourfold higher rate of congenital abnormalities compared to seropositive women [11]. Timing of primary maternal infection during pregnancy is an important prognosticating factor of fetal consequences, with long-term neonatal sequalae being unique to mothers with primary infection in the first trimester [12]. Yves Ville and colleagues estimated that up to 57% of infected neonates from primary maternal CMV infection in the first trimester of pregnancy would develop long-term sequalae [13].

CMV seroprevalence varies between different countries, typically being high in developing countries and lower in developed countries. CMV IgG seropositivity in pregnant women ranges from 61% in France [14] to nearly 100% in developing countries like Brazil [15]. There is strong epidemiological evidence showing causal link between hygiene measures and infections [16]. Better standard of cleanliness and higher socioeconomic status in developed countries have positive effects on delaying exposure to CMV.

Studies on CMV seroprevalence in Singapore are limited. The last published data on CMV seroprevalence in Singapore antenatal population was reported by Wong et al. from a small cohort of 120 patients using older enzyme-linked immunosorbent assay more than 20 years ago [17]. In their study, CMV IgG positivity was observed in 87% of pregnant women in 1998, which is a much higher seroprevalence expected from an industrialized country. Since then, there has been no published reports on CMV IgG positivity in pregnant women in Singapore for the last two decades.

The aim of this study is to examine changes in CMV seroprevalence in antenatal populations in Singapore. We hypothesize that CMV IgG seropositivity in Singaporeans has changed with improving socioeconomic conditions over the past several decades, with current figures resembling that of other developed countries. Women who are seronegative at the start of their pregnancy are at risk of primary CMV infection, for whom interventions may help reduce vertical transmission and prevent congenital CMV disease. Data on the most updated CMV seroprevalence in pregnant women in Singapore is an important epidemiological tool to determine the value of implementing universal screen for CMV.

## Materials and methods

### Data collection and analysis of sample

Pregnant women aged 21–60 who received perinatal care and had routine antenatal screening blood tests conducted within the first trimester of pregnancy at the Obstetrics Department of Singapore General Hospital between 1 January 2021 and 1 August 2021 were included. In our hospital, serum samples collected at this ‘booking visit’ are stored for 12 months. Women with insufficient stored sera for serology testing were excluded from the study.

Participants were randomly selected from a comprehensive list using a computer-generated random number sequence. 385 samples were sent to an independent party, the Singhealth Tissue Repository–SGH Satellite Bank for de-identification and subsequently tested for CMV IgG by chemiluminescent microparticle immunoassay. The manufacturer reported a sensitivity of 100% with 99.15% specificity for this assay. The presence of CMV-specific IgG (positive result interpreted according to the manufacturer’s specifications) indicates past exposure prior to pregnancy.

### Statistical analysis

A sample size of 385 was determined based on assumed seroprevalence of 60%, with a desired precision of 5% and a 95% confidence interval, to achieve 80% power for detecting this seroprevalence rate while maintaining a Type 1 error rate of 5%. Data were entered and analysed using the statistical software SPSS 28.0 (IBM Corp., Armonk, NY, USA). The relationship between age and CMV seropositivity was analysed by the Pearson’s χ^2^ test. A *p* value of < 0.05 was considered statistically significant. Statistical analysis was carried out with the assistance of the institute’s biostatistician.

This study was supported by the Singhealth RHS PULSES Grant and was granted a waiver of consent for research by the Singhealth Centralised Institutional Review Board as involves analysis of de-identified blood samples collected from routine clinical procedures, posing no more than minimal risk to participants.

## Results

The participants’ ages ranged from 19 to 44 years (median age 32). Two (0.5%) women were less than 20 years, 120 (31.2%) were between 20 and 29 years, 244 (63.4%) were in 30–39 years, and 19 (4.9%) were more than 40 years.

### Overall CMV seropositivity in our antenatal population

CMV IgG was positive in 276 women and negative in 109 women. Thus, overall, 71.7% of pregnant women in our study were CMV IgG seropositive.

### Seroprevalence of CMV by age groups

Except in the youngest age group, CMV IgG positivity trend was noted to increase with age; where it was 100% in women < 20 years, 68.3% in the 20–29-year age group, 72.5% in the 30–39-year age group, and 79.0% in the above 40 years (Table 1).

**Table 1:** CMV IgG seropositivity by age groups

	Category	Total tested	CMV IgG		95% CI	***p*** value
			Positive	Prevalence (%)		
Age (median age, yr)	< 20 (19) 20–29 (27) 30–39 (33) ≥ 40 (40)	2 120 244 19	2 82 177 15	100 68.3 72.5 78.9	0.34, 1.00 0.60, 0.76 0.66, 0.78 0.57, 0.92	0.157 < 0.001 < 0.001 0.012
Total		385	276	71.7	0.67, 0.76	< 0.001

## Discussion

This is the first study in over two decades to provide important epidemiological data on the seroprevalence of CMV amongst pregnant women in Singapore. Our data estimated 71.7% of our antenatal population to be seropositive for CMV IgG. This is 15.3% lower than the seropositivity rate for CMV IgG reported by Wong and colleagues in 1998 [17] and supports our initial hypothesis that the proportion of women seropositive for CMV IgG is likely to have fallen with improved hygiene and socioeconomic conditions in Singapore.

This seroprevalence rate is higher than that reported in France [14], similar to Italy [18] and Finland [19], and lower than countries like Japan [20], China [21], Romania [22], and Pakistan[23], where seroprevalence rates close to 100% have been reported (Table 2). The chronologic change of CMV seroprevalence observed in our country is similar to that reported in other countries, such as Spain and Germany, which observed statistically significant decreases in IgG positivity in their population over the past decade [24, 25]. Interestingly, this change has not been universal among developed countries, even within Asia. South Korea continued to report a high seroprevalence of CMV at 94.1% between 2006 and 2015, without significant change in CMV seropositivity since 1995 [26].

**Table 2:** CMV seroprevalence in pregnant women of different countries

	Leruez-Ville et al. 2017 [14]	Trombetta et al. 2021 [18]	Our study, 2021	Kaneko et al. 2023 [20]	Waseem et al. 2017 [23]
Country	France	Italy	Singapore	Japan	Pakistan
Number of participants (n)	2378	360	385	1163	172
CMV seroprevalence in antenatal population (%) (95% CI)	61 (0.58, 0.62)	70.8 (0.66, 0.76)	71.7 (0.67, 0.76)	82.5 (not provided)	99.4 (not provided)

Wong and colleagues [17] previously studied the influence of age on CMV seropositivity and reported an increased seroprevalence in women above 30 years of age (80.8% for age > 30 *vs* 76.5% for age ≤ 30). This trend has persisted, our study found a seroprevalence of 73.0% in women aged 30 years and older versus 68.9% in those under 30 years of age (P = 0.66). The 100% CMV IgG positivity reported in the < 20-year age group is unlikely to be representative of the seroprevalence in that age group as only two samples were available for analysis in that cohort.

A declining trend in CMV seropositivity in women of reproductive age is important from a public health standpoint. As larger proportion of women will now be at risk of acquiring primary CMV infection during pregnancy, primary prevention efforts will be more important. Our data suggests that more than a quarter (109/385, 28.3%) of women were seronegative for CMV at the start of their pregnancy.

Primary CMV infections have a higher risk of vertical transmission, symptomatic congenital infection and more severe sequelae compared to non-primary infections [27]. These incidences can be reduced through preventative measures such as hygiene interventions [28, 29]. Examples include avoiding contact with infected bodily fluids (e.g., saliva, tears and urine), hand hygiene and regular cleaning of potential fomites such as diaper change areas and toys [30]. These measures need to be undertaken during the preconception period and throughout pregnancy and are particularly important for women living in households with young children as they are a frequent source of CMV and can shed the virus for extended periods after infection [31].

The lack of public health education on CMV is evidence from a local study published by Lim and colleagues, who reported that only 20% of pregnant women attending antenatal care were aware of CMV [32]. As a larger proportion of pregnant women are now at risk of primary CMV infections, there is an urgent need for public health interventions that focus on improving prenatal education and promoting behavioural modifications to reduce the risk of CMV acquisition.

Although a lower seroprevalence of CMV in pregnant women suggest an increased risk of primary maternal infection during pregnancy, this does not necessarily translate to a rising incidence of congenital CMV. Lower seroprevalence is often associated with improved hygiene and higher socioeconomic condition, which are typically maintained throughout pregnancy. Nonetheless, it is crucial to maintain these enhanced hygiene standards, as seronegative women at the onset of pregnancy remain at significant risk of delivering CMV-infected newborns, which further emphasizes the importance of continued public health education and implementation of robust public health measures to effectively manage and reduce CMV acquisition rates among seronegative pregnant women.

In countries where seropositivity is declining, the more critical factor is the shift in proportion of congenital CMV cases resulting from primary versus non primary infections. As more women enter pregnancy being seronegative, a higher proportion of congenital CMV cases arise from primary infections, which carry a greater risk of vertical transmission, having symptomatic infants at birth, and worse neurological sequalae compared to nonprimary infections. For instance, in France, where maternal seroprevalence is 61%, 52% of congenital CMV cases at birth are due to primary infections. This is in contrast with regions with near universal seroprevalence, where almost all cases of congenital CMV are due to nonprimary infections, which are typically associated with less severe outcomes.

As the number of seronegative women entering pregnancy increases, secondary prevention measures, such as antenatal screening and treatment, are becoming increasingly crucial. Recent epidemiological data from two French maternity hospitals in Paris reveal that seronegative women are four times more likely to deliver an infected newborn compared to their seropositive counterparts [14]. Universal antenatal serological screening can be done in the first trimester to detect periconceptional and first trimester primary infections that pose the highest risk of fetal sequalae [13]. Recent advancement in antenatal treatment for primary CMV infection have significantly altered this field. Notably, data from Shahar-Nissan and colleagues has suggested that daily administration of 8 g of valaciclovir in women with primary CMV infection acquired early in pregnancy resulted in a 71% reduction in the rate of fetal transmission when compared to placebo [33]. A recent systematic review similarly concluded that prenatal valacyclovir administration with maternal primary CMV infection reduces the risk of congenital CMV infection, with a low risk of reversible acute renal failure [34].

In terms of cost, cost-effectiveness studies on screening for congenital CMV infection have shown the prevalence of congenital CMV infection to be an important factor influencing incremental cost-effectiveness ratios [35]. Offering screening therefore may become more cost-effective in light of these updated lower seroprevalence rates where a larger proportion of women are at risk. Further studies evaluating the cost-effectiveness of universal maternal CMV screening with subsequent antenatal valacyclovir treatment in the local context are needed.

In conclusion, the seroprevalence of CMV in pregnant women has decreased compared to 20 years ago. As a larger proportion of pregnancies are now at risk of congenital CMV, primary and secondary prevention efforts to reduce the incidence of primary CMV infections during pregnancy will become more important. This study provides a much-needed update on the changing CMV seroprevalence in the antenatal population in Singapore, more than 20 years after the first study of its kind. The sample size of this study is also three times larger than that of the previous study. We acknowledge the study’s limitation in terms of its single-centre nature; nevertheless, it remains representative of the national data as samples were randomly selected and include the varying demographics of pregnant women in Singapore. A multicenter cohort study with a larger number of participants will be helpful to provide a better picture on the overall CMV seroprevalence in the antenatal population in Singapore.

## Data Availability

The datasets used during the current study are available from the corresponding author upon reasonable request.
